# The geography of post-disaster mental health: spatial patterning of psychological vulnerability and resilience factors in New York City after Hurricane Sandy

**DOI:** 10.1186/s12942-015-0008-6

**Published:** 2015-06-10

**Authors:** Oliver Gruebner, Sarah R Lowe, Laura Sampson, Sandro Galea

**Affiliations:** Department of Epidemiology, Columbia University, Mailman School of Public Health, New York, NY USA; Department of Epidemiology, Boston University, School of Public Health, Boston, MA USA

**Keywords:** Natural disasters, Mental health, Urban, Spatial epidemiology, Moran’s I, Spatial regime

## Abstract

**Background:**

Only very few studies have investigated the geographic distribution of psychological resilience and associated mental health outcomes after natural or man made disasters. Such information is crucial for location-based interventions that aim to promote recovery in the aftermath of disasters. The purpose of this study therefore was to investigate geographic variability of (1) posttraumatic stress (PTS) and depression in a Hurricane Sandy affected population in NYC and (2) psychological vulnerability and resilience factors among affected areas in NYC boroughs.

**Methods:**

Cross-sectional telephone survey data were collected 13 to 16 months post-disaster from household residents (N = 418 adults) in NYC communities that were most heavily affected by the hurricane. The Posttraumatic Stress Checklist for DSM-5 (PCL-5) was applied for measuring posttraumatic stress and the nine-item Patient Health Questionnaire (PHQ-9) was used for measuring depression. We applied spatial autocorrelation and spatial regimes regression analyses, to test for spatial clusters of mental health outcomes and to explore whether associations between vulnerability and resilience factors and mental health differed among New York City’s five boroughs.

**Results:**

Mental health problems clustered predominantly in neighborhoods that are geographically more exposed towards the ocean indicating a spatial variation of risk within and across the boroughs. We further found significant variation in associations between vulnerability and resilience factors and mental health. Race/ethnicity (being Asian or non-Hispanic black) and disaster-related stressors were vulnerability factors for mental health symptoms in Queens, and being employed and married were resilience factors for these symptoms in Manhattan and Staten Island. In addition, parental status was a vulnerability factor in Brooklyn and a resilience factor in the Bronx.

**Conclusions:**

We conclude that explanatory characteristics may manifest as psychological vulnerability and resilience factors differently across different regional contexts. Our spatial epidemiological approach is transferable to other regions around the globe and, in the light of a changing climate, could be used to strengthen the psychosocial resources of demographic groups at greatest risk of adverse outcomes pre-disaster. In the aftermath of a disaster, the approach can be used to identify survivors at greatest risk and to plan for targeted interventions to reach them.

## Background

Hurricane Sandy made landfall in the greater New York City area on October 29, 2012. A combination of warm Caribbean air, a high-pressure system over Greenland, a disturbance in the jet stream and a spring tide due to a full moon caused a storm surge of more than 14 feet to hit the coastline of New Jersey and New York City, one of the most densely populated areas of the United States. The storm caused 43 deaths and contributed to $19 billion in damage in NYC alone [1-3]. Around 63,000 houses were damaged and 300 destroyed. Thousands of residents were left without power, and experienced infrastructural damage (e.g., to public transportation and hospitals) and limited access to necessary resources, including food, water, and healthcare [4].

The literature on disaster mental health has been growing rapidly in recent years and we now have a good understanding of the factors associated with common mental health conditions that have been found to be elevated in the post-disaster period, including posttraumatic stress disorder (PTSD) and major depression [5-8]. Research to date has documented a variety of *vulnerability* and *resilience factors* that are positively and negatively associated with these post-disaster mental health symptoms, respectively [9]. Robust vulnerability factors include socioeconomic disadvantage and higher levels of exposure to disaster-related stressors and traumatic events, whereas resilience factors include higher socioeconomic status, social support, and favorable living arrangements (e.g., being married versus single) [10,11]. Psychological outcomes in the aftermath of disasters are likely to vary across space, such that high and low levels of symptoms are concentrated in specific geographic areas. Additionally, the extent to which vulnerability and resilience factors are associated with post-disaster psychological responses is likely to vary across geographic regions.

Therefore, there is potential to use geospatial and spatial epidemiological analyses to better understand the distribution of psychological outcomes, and variation in the strength of vulnerability and resilience factors, in the aftermath of disasters [12,13]. However, only three studies to our knowledge have included geographic analyses in their studies on post-disaster mental health so far. A study by Curtis et al. [14] presented maps of potential vulnerability factors in the aftermath of Hurricane Katrina to show which areas of New Orleans might have had the most severe post-hurricane stress-related health outcomes. Two other studies used a spatial approach to show that greater proximity to the disaster was associated with higher psychiatric symptoms [15,16].

There remains much that we do not know about the spatial distribution of psychological vulnerability and resilience after disasters. First, we have limited understanding of the geographic patterning of the mental health consequences of disasters. Knowledge about the extent of spatial clustering and the locations of clusters could inform practitioners about which geographic areas might be in greatest need of post-disaster services. Second, we are not aware of work that has systematically assessed whether associations between vulnerability and resilience factors and post-disaster mental health vary across different geographic regions. With regard to changes in average climate conditions around the globe and expected extreme events (e.g., hurricanes, heat waves, droughts, or floods) [17,18], such findings could have important implications for tailoring interventions to specific communities based on the pertinent vulnerability and resilience factors. With these gaps in the literature in mind, we set out to answer two core questions: (1) Are posttraumatic stress (PTS) and depression among Hurricane Sandy affected New York City residents spatially clustered at the individual level?; and (2) Do associations between vulnerability and resilience factors and mental health outcomes vary across New York City’s five boroughs?

## Results

### Spatial clusters of mental health

#### Global spatial clustering

We found weak but statistically significant global spatial clustering of PTS in all tested neighborhood definitions, i.e. k-nn and fixed distance bands, with slightly decreasing autocorrelation when increasing the number of neighbors or distances between the neighbors. Peaks were found for 4 km (Moran’s I: 0.06, z-value: 4.12, p-value: <0.001), 7.5 km (0.06, 6.23, <0.001), 9 km (0.05, 6.85, <0.001), and 15 km (0.04, 9.6, <0.001). For depression, we found negligible global spatial clustering with the fixed distance bands starting from 9 km (Moran’s I: 0.01, z-value: 1.8, p-value: 0.04) onwards. Peaks were found for 13 km (0.01, 2.09, 0.04) and for 16 km (0.01, 3.17, 0.002). We proceed with reporting only the 9 km fixed distance band in the remainder of this paper since this neighborhood definition showed the highest z-value in the Moran’s I range above 0.05 for PTS and since this was comparable to depression.

### Local spatial clusters

We identified the names of neighborhoods wherein there were spatial clusters by consulting the NYC Department of City Planning website for neighborhood definitions (http://www.nyc.gov/html/dcp/html/neighbor/index.shtml). Using 9 km fixed distance bands, we found that high values of PTS were locally clustered around six neighborhoods in Queens: Howard Beach, Brookville, Far Rockaway, Arverne, Rockaway Park and Breezy Point. Further clusters were found around the following neighborhoods in Brooklyn: Starrett City, Canarsie, Bergen Beach, and Sheepshead Bay.

**Figure 1:**
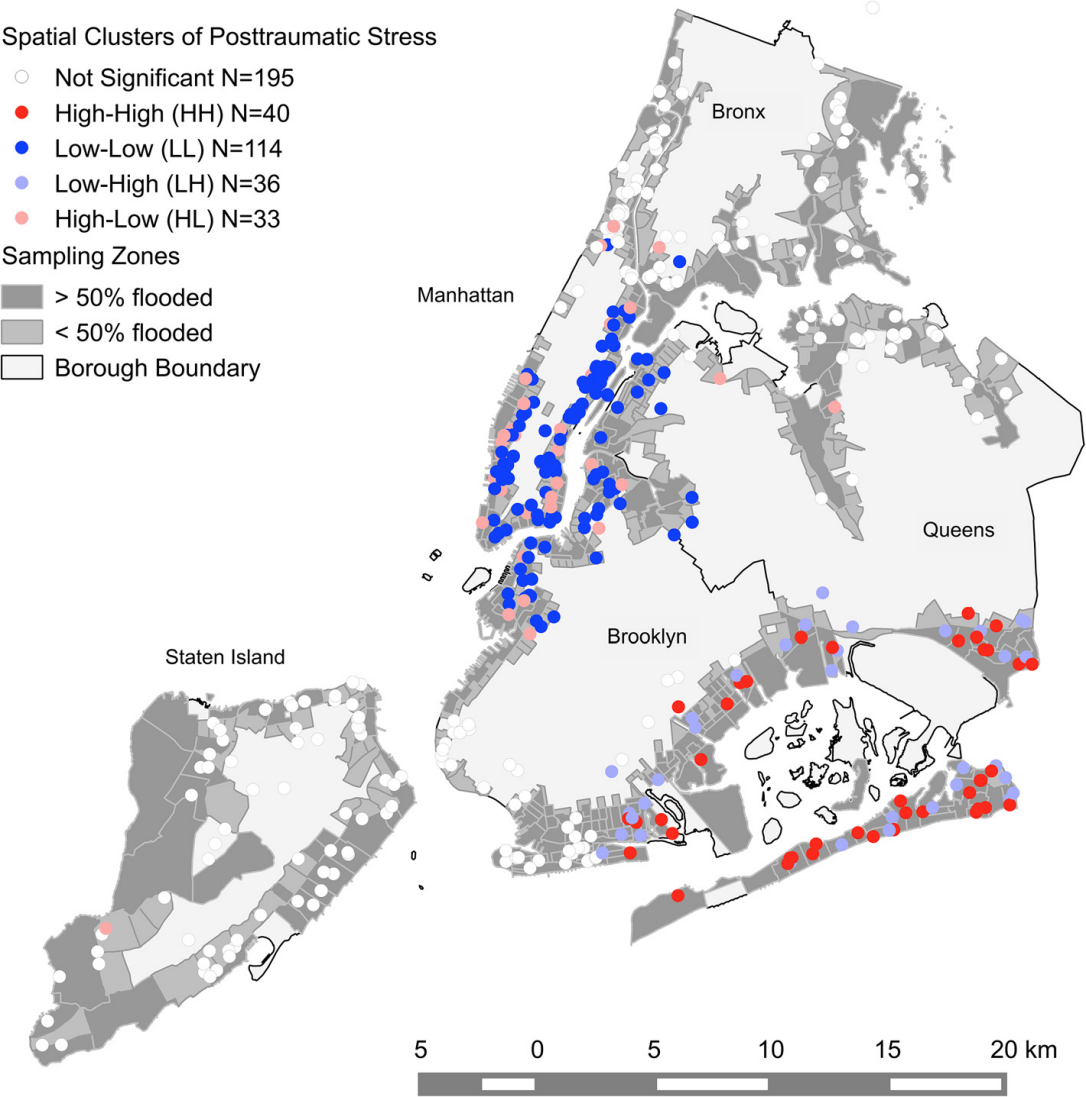
Spatial clusters of PTS. Each dot on the map indicates a respondent’s location. The map indicates significant (p < 0.05) spatial clusters of high (HH) or low (LL) PTS. High values surrounded by low values (HL) and vice versa (LH) indicate outliers. A fixed distance of 9 km was used in the statistics. Note that a geometric shift of approx. 100 m has been introduced for displaying the point data so that single houses may not be identified for privacy reasons.

Low values of PTS were spatially clustered in all sampled neighborhoods south of Central Park, the Upper Eastside and East Harlem in Manhattan, Roosevelt Island, Astoria, Hunters Point, and Ridgewood in Queens, and Greenpoint, Williamsburg, Brooklyn Heights, Carroll Gardens, and Park Slope in Brooklyn. Since our aim was not to identify spatial outliers (i.e., clusters of High-Low and Low-High symptom levels), we do not report them here, although they are presented on the map (cf. Figure 1).

**Figure 2:**
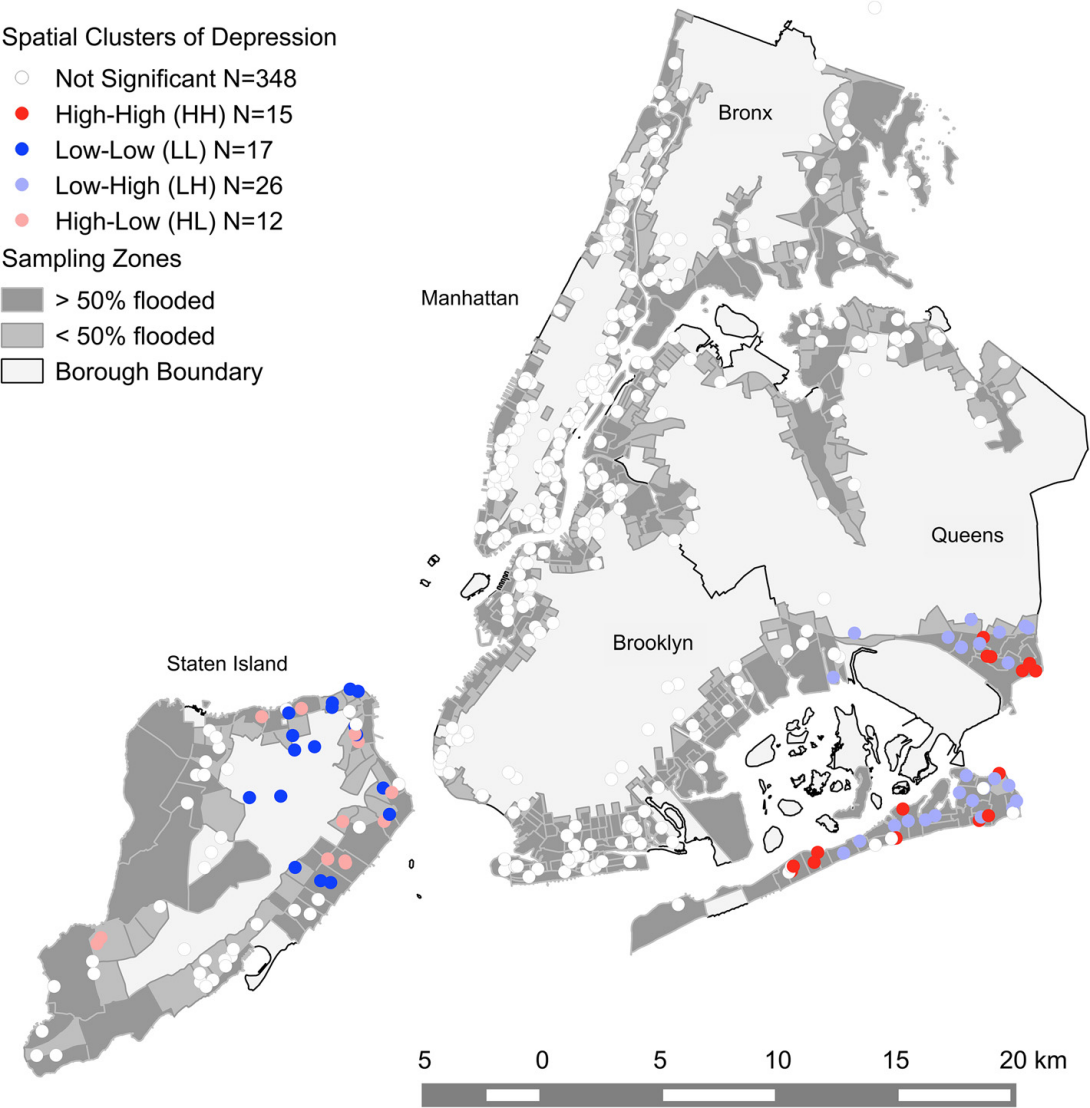
Spatial clusters of depression. Each dot on the map indicates a respondent’s location. The map indicates significant (p < 0.05) spatial clusters of high (HH) or low (LL) depression. High values surrounded by low values (HL) and vice versa (LH) indicate outliers. A fixed distance of 9 km was used in the statistics. Note that a geometric shift of approx. 100 m has been introduced for displaying the point data so that single houses may not be identified for privacy reasons.

High values of depression were clustered around Brookville, Far Rockaway, and Arverne in Queens. In contrast, low values of depression were clustered around New and West Brighton, Fort Wadsworth, Egbertville, and Willowbrook in Staten Island. Since our aim was not to identify spatial outliers (i.e., clusters of High-Low and Low-High symptom levels), we do not report them here, although they are presented on the map (cf. Figure 2).

### Geographic variability of associations between explanatory variables and mental health

As seen in Table 1, levels of PTS and depression varied between affected areas within boroughs with higher than above average symptom levels in areas within Queens, followed by the Bronx and Brooklyn. PTS was lowest in areas within Manhattan, followed by Staten Island. In contrast, depression was lowest in affected areas of Staten Island, followed by Manhattan.

**Table 1:** **Means and frequencies for all variables included in the study across New York City boroughs**

	**NYC**		**Manhattan**		**Bronx**		**Brooklyn**		**Queens**		**Staten Island**	
	**M/% (SE)**	**N**	**M/% (SE)**	**N**	**M/% (SE)**	**N**	**M/% (SE)**	**N**	**M/% (SE)**	**N**	**M/% (SE)**	**N**
Age	50.23 (.84)	--	49.95 (1.59)	--	48.54 (3.07)	--	49.53 (1.76)	--	50.93 (1.74)	--	52.46 (1.99)	--
Female	62.2% (.02)	260	55.2 (.04)	69	64.9% (.08)	24	59.4% (.05)	57	68.8% (.05)	64	68.7% (.06)	46
Asian	5.3% (.01)	22	9.6% (.03)	12	2.7% (.03)	1	4.2% (.02)	4	3.2% (.02)	3	3% (.02)	2
Non-Hispanic Black	18.7% (.02)	78	13.6% (.03)	17	40.5% (.08)	15	15.6% (.04)	15	29% (.05)	27	6% (.03)	4
Hispanic	19.1% (.02)	80	31.2% (.04)	39	29.7% (.08)	11	8.3% (.03)	8	15.1% (.04)	14	11.9% (.04)	8
Other ethnicity	6.5% (.01)	27	7.2% (.02)	9	8.1% (.05)	3	8.3% (.03)	8	5.4% (.02)	5	3% (.02)	2
High school education or less	25.4% (.02)	106	21.6% (.04)	27	37.8% (.08)	14	21.9% (.04)	21	24.7$ (.05)	23	31.3% (.06)	21
Employed	54.8% (.02)	229	56.8% (.05)	71	45.9% (.08)	17	55.2% (.05)	53	60.2% (.05)	56	47.8% (.06)	32
Married or cohabitating	43.5% (.02)	182	36.8% (.04)	46	45.9% (.08)	17	41.7% (.05)	40	50.5% (.05)	47	47.8% (.06)	32
Parent, living with child at time of Sandy	21.8% (.02)	91	14.4% (.03)	18	24.3% (.07)	9	24% (.04)	23	23.7% (.04)	22	28.4% (.06)	19
Experienced or witnessed trauma in addition to Sandy	49.3% (.03)	206	53.6% (.05)	67	23.2% (.08)	16	51% (.05)	49	48.4% (.05)	45	43.3% (.06)	29
Number of Sandy-related trauma	.06 (.01)	--	.05 (.02)	--	.05 (.04)	--	.1 (.04)	--	.05 (.03)	--	.05 (.03)	--
Number of Sandy-related stressors	.64 (.05)	--	.02 (.05)	--	.29 (.09)	--	.73 (.11)	--	.12 (.15)	--	.69 (.12)	--
Posttraumatic stress (PCL-5 severity score)	7.12 (.55)	--	5.12 (.76)	--	7.81 (1.93)	--	7.75 (1.43)	--	9.61 (1.33)	--	6.08 (.93)	--
Depression (PHQ-9 severity score)	3.25 (.23)	--	3.06 (.39)	--	3.46 (.94)	--	3.28 (.49)	--	3.99 (.55)	--	2.42 (.49)	--
Total N		418		125		37		96		93		67

PCL-5 = Posttraumatic Stress Checklist for DSM-5. PHQ-9 = Patient Health Questionnaire-9.

### Global associations between explanatory variables and mental health across the boroughs

Globally, i.e., ignoring any variation across boroughs, we found that older age, being Hispanic or non-Hispanic Black, having a high school education or less, reporting trauma in addition to Hurricane Sandy, and having more hurricane-related stressors were significantly associated with higher PTS. Hence we identified these characteristics as vulnerability factors for PTS. Similarly, we found that having a high school education or less, reporting trauma in addition to Sandy, and experiencing more hurricane-related stressors were significant vulnerability factors for depression (cf. Table 2).

**Table 2:** **Global OLS model for associations between explanatory variables and mental health outcomes**

**Explanatory variables/mental health outcomes**	**PTS**			**DEPRESSION**		
	**Estimate**	**Sig.**	**S.E.**	**Estimate**	**Sig.**	**S.E.**
Age	.08	*	.03	.00		.01
Female	1.45		1.05	.25		.48
Asian	1.31		2.21	.20		1.02
Non-Hispanic Black	4.37	**	1.32	.84		.61
Hispanic	3.54	**	1.34	.75		.62
Other ethnicity	−1.26		2.04	.63		.94
High school education or less	3.40	**	1.22	1.33	*	.56
Employed	−1.64		1.06	−.77		.49
Married or cohabiting	−1.85	.08	1.05	−.53		.48
Parent, living with child at time of Sandy	1.48		1.28	.21		.59
Experienced or witnessed trauma in addition to Sandy	3.16	**	1.02	1.63	***	.47
Number of hurricane-related stressors	3.73	***	.49	.94	***	.23
Number of hurricane-related trauma	1.53		1.93	.69		.89
**Model fit**						
Adjusted R2:	.23			.09		
AIC:	3,116.22			2,468.83		

N = 418. Significance level: <0.001***, <0.01**, <0.05*.

### Geographic variations of associations between explanatory variables and mental health between the boroughs

We applied a spatial regimes approach and used the same neighborhood fixed distance of 9 km found above to account for spatial heterogeneity of explanatory variables. Since the residuals from the OLS model with regimes for PTS were spatially autocorrelated (Lagrange multiplier test of lag type: 4.51, p <0.05), we applied a spatial lag regimes model. For a better comparability to PTS, we also applied a spatial lag model for depression, even though the residuals from this model were not spatially autocorrelated. The overall model fit (R^2^) for both PTS and depression models could be improved by 10% when compared to the non-spatial OLS models with regimes, and 34% and 24% of the variance could be explained, respectively (cf. Tables 3 and 4).

**Table 3:** **Geographic variability of associations between explanatory variables and PTS between the five NYC boroughs**

	**Manhattan**		**Bronx**		**Brooklyn**		**Queens**		**Staten Island**		**Chow test**
	**N = 125**		**N = 37**		**N = 96**		**N = 93**		**N = 67**		**N = 5**
	**Est.**	**S.E.**	**Est.**	**S.E.**	**Est.**	**S.E.**	**Est.**	**S.E.**	**Est.**	**S.E.**	**Est.**
Age	.05	.04	−.12	.12	.22**	.09	.05	.06	.03	.06	6.23
Female	1.09	1.46	4.25	4.37	−2.19	3.48	.15	1.85	3.77*	1.80	3.59
Asian	−2.12	1.27	12.19***	3.65	3.58	5.29	16.81	9.98	−2.68	2.35	18.16**
Non-Hispanic Black	−.11	2.15	−.56	3.58	7.65	5.57	8.69**	3.27	.36	7.20	6.43
Hispanic	4.64***	1.76	5.22	4.05	3.83	5.27	.22	2.37	3.27	2.46	2.51
Other race/ethnicity	−2.26	2.39	−3.54	4.72	−3.53	3.00	.11	3.55	8.67	7.82	2.59
High school education or less	4.96*	2.00	2.12	3.54	5.08	4.16	3.26	2.74	2.63	2.07	1.00
Employed	−2.67*	1.35	−5.66	4.32	−1.44	2.76	−.02	2.42	−.95	1.52	2.08
Married or cohabiting	−2.52 (.06)	1.33	−2.52	3.42	−2.51	2.43	−.99	2.29	−3.87*	1.72	1.04
Parent, living with child at time of Sandy	1.66	1.81	−8.08*	3.81	3.60	2.92	3.97	3.37	1.91	2.05	7.40
Experienced or witnessed trauma in addition to Sandy	3.66**	1.40	8.02***	2.32	−.27	2.76	4.58	2.83	.31	1.62	9.38 (.05)
Number of hurricane-related stressors	2.14*	.87	4.13	3.11	4.13*	1.73	4.37***	1.08	3.36**	1.03	2.64
Number of hurricane-related trauma	.66	2.62	1.94	5.30	1.06	5.40	2.72	3.96	5.62	4.05	1.12
**Global spatial lag**											
Spatially lagged PTS across all boroughs	−0.47	.36									
**Model fit**											
Pseudo R2	.35										
Spatial Pseudo R2	.34										
**Global structural stability test**											
Chow test	120.66***										
**Spatial dependence**											
Anselin-Kelejian test	1.28										

N = Number of cases, Est. = Coefficient estimate, S.E. = Standard error, Significance level: <0.001***, <0.01**, <0.05*. P-value reported in brackets when marginal significant. Spatial neighborhood definition: 9 km, N total = 418.

**Table 4:** **Geographic variability of associations between explanatory variables and depression between the five NYC boroughs**

	**Manhattan**		**Bronx**		**Brooklyn**		**Queens**		**Staten Island**		**Chow test**
	**N = 125**		**N = 37**		**N = 96**		**N = 93**		**N = 67**		**N = 5**
	**Est.**	**S.E.**	**Est.**	**S.E.**	**Est.**	**S.E.**	**Est.**	**S.E.**	**Est.**	**S.E.**	**Est.**
Age	-.02	.02	-.09	.06	.04	.03	-.01	.03	.01	.03	6.08
Female	-.31	.81	1.07	1.86	.50	1.21	−1.07	1.08	2.09*	1.02	5.36
Asian	-.42	1.32	.28	1.43	-.96	.94	7.10**	2.48	−1.25	.97	10.48*
Non-Hispanic Black	-.24	1.03	−2.11	2.20	2.01	1.62	1.82	1.36	−1.62	1.79	4.67
Hispanic	1.55	.92	−1.71	2.57	1.29	1.70	.74	1.69	.37	1.65	1.68
Other race/ethnicity	-.39	1.54	3.28	2.44	1.80	2.33	-.07	2.61	3.39	2.00	3.28
High school education or less	1.08	1.10	4.74*	2.30	-.01	1.33	1.46	1.37	2.01	1.19	3.59
Employed	−1.67	.95	.45	2.28	−1.68	1.01	-.26	1.12	.36	.89	3.79
Married or cohabiting	-.40	.81	1.20	1.95	−1.07	.87	-.51	1.08	−2.74***	.80	6.56
Parent, living with child at time of Sandy	-.60	.96	−5.61**	2.18	2.52*	1.18	1.56	1.31	-.10	.88	12.91*
Experienced or witnessed trauma in addition to Sandy	2.07**	.79	2.14	1.85	.92	.95	2.11 (.07)	1.17	-.07	.80	4.62
Number of hurricane-related stressors	.75	.79	.81	1.45	.76	.59	1.34**	.41	0.95 (.07)	.52	1.03
Number of hurricane-related trauma	−1.38	.88	6.34*	2.95	.65	1.56	.04	1.27	8.63**	2.71	17.37**
**Global spatial lag**											
Spatially lagged depression across all boroughs	-.39	.7									
**Model fit**											
Pseudo R2	.25										
Spatial pseudo R2	.24										
**Global structural stability test**											
Chow test	103.82***										
**Spatial dependence**											
Anselin-Kelejian test	.64										

N = Number of cases, Est. = Coefficient estimate, S.E. = Standard error, Significance level: <0.001***, <0.01**, <0.05*. P-value reported in brackets when marginal significant. Spatial neighborhood definition: 9 km, N total = 418.

The association between vulnerability/resilience factors and each mental health outcome varied between the boroughs, as reflected in model and coefficient heterogeneity (cf. Chow test in Tables 3 and 4). The Chow tests for coefficients’ heterogeneity for PTS response were significant for Asian ethnicity and reporting trauma in addition to Hurricane Sandy. Coefficients for depression varied significantly among the boroughs for Asian ethnicity, being a parent, and number of hurricane-related trauma. Although the coefficients for other vulnerability and resilience factors did not reach statistical significance with the Chow test, we took note of variation in the extent to which each was associated with PTS and depression across the five boroughs.

#### Demographic characteristics

In addition to Asian ethnicity, we also noted that associations between older age, female gender, Hispanic ethnicity, and being non-Hispanic Black and post-disaster mental health problems also varied across the boroughs (cf. Tables 3 and 4). Older age was positively associated with PTS in Brooklyn and female gender was positively associated with both PTS and depression in Staten Island. Being Hispanic was associated with higher PTS in Manhattan, Asian in the Bronx, and non-Hispanic black in Queens. Being Asian was also positively associated with depression in Queens.

#### Socioeconomic characteristics

Socioeconomic factors were found to be both vulnerability and resilience factors for post-disaster mental health symptoms. Having high school education or less was associated with higher PTS in Manhattan and higher depression in the Bronx. In contrast, being employed was associated with lower PTS in Manhattan.

#### Living arrangements

Being married or cohabiting with a partner was a resilience factor for PTS in Manhattan (*p* = 0.06) and for both PTS and depression in Staten Island. In contrast, parents living with a child at the time of Sandy had significantly higher depression than non-parents in Brooklyn. In the Bronx, however, being a parent was a resilience factor for both PTS and depression.

#### Pre-hurricane exposure

Those who experienced or witnessed trauma in addition to Sandy had higher PTS in the Bronx, depression in Queens (*p* = 0.07), and both PTS and depression in Manhattan.

#### Sandy-related exposure

Exposure to a higher number of hurricane-related stressors was a vulnerability factor for PTS in Manhattan, Brooklyn, Queens, and Staten Island. It was also a vulnerability factor for depression in Queens, as well as in Staten Island (*p* = 0.07). Respondents with a higher number of hurricane-related traumas had higher depression in the Bronx and Staten Island.

## Discussion

We sought to identify spatial clusters of PTS and depression among residents of New York City neighborhoods that were affected by Hurricane Sandy. We also aimed to explore whether psychological vulnerability and resilience factors varied among the five New York City boroughs. We found that mental health outcomes were spatially clustered in neighborhoods within the boroughs, with local clusters of high PTS in neighborhoods within Queens and Brooklyn and clusters of low PTS in neighborhoods within Manhattan and also within Queens and Brooklyn. High depression clustered spatially in neighborhoods within Queens and low depression clustered in neighborhoods on Staten Island. In addition, we found variation in vulnerability and resilience factors for higher post-disaster PTS and depression among the boroughs. In particular, we found significant variation in associations between demographic characteristics (e.g., age, race/ethnicity), living arrangements (e.g., parent status), and exposure to hurricane-related trauma and mental health outcomes across the five boroughs.

Our analysis provided evidence that there were spatial clusters of both high and low PTS and depression within the study area, indicating geographic variation in the risk for post-disaster mental health problems across the boroughs (all but the Bronx). Notably, the hotspots of PTS and depression did not overlap entirely. This could be due in part to differences in how they were assessed, with PTS assessed specifically in reference to the hurricane and its aftermath, and depression assessed more generally. As such, the spatial patterns of PTS could be seen as more hurricane-specific, whereas those of depression could reflect both the impact of the hurricane and ongoing mental health problems. In interpreting these results, it is important to note that these findings are unlikely to be due to local spillover among the respondents in the study, as in the context of a large, urban area like NYC it is unlikely that the respondents were interacting with each other. Rather, it is more likely that shared vulnerability and resilience factors among residents of the same neighborhoods account for the spatial clusters we observed. For example, we found spatial clusters of above average mental health outcomes in neighborhoods that are geographically more exposed towards the ocean (Southeastern Queens and Brooklyn) assuming that vulnerability factors were more pronounced in those areas [1,19]. In contrast, resilience factors could have played a crucial role in shaping the clusters of lower than average mental health outcomes in neighborhoods within Manhattan and Northwestern Brooklyn and Queens. However, the large number of census tracts represented and the small number of respondents within each of them prohibited exploration of variation in vulnerability and resilience factors across census tracts. Instead, we explored spatial variation in associations between vulnerability and resilience factors and mental health across the five NYC boroughs. There was significant variation in the extent to which Asian ethnicity and trauma exposure in addition to Hurricane Sandy were associated with PTS, and Asian ethnicity, parent status, and hurricane-related trauma were associated with depression. Although variation in the coefficients for other vulnerability and resilience factors across the boroughs did not reach statistical significance, we observed several other instances in which factors were predictive of outcomes in one or more, but not all, of the boroughs.

Furthermore, we noted that some vulnerability and resilience factors that were not significant predictors of mental health outcomes in the full sample reached statistical significance within one or more of the boroughs. For example, Asian ethnicity was a significant vulnerability factor for higher depression in Queens and for higher PTS in the Bronx. On the other hand, factors that reached statistical significance in the full sample were not significant predictors across all of the boroughs. We found, for example, that exposure to more hurricane-related stressors, a robust predictor of both higher PTS and depression in the full sample and in prior research (e.g., [10, 11]), was not associated with PTS in the Bronx or with depression in Queens. Finally, the results showed that a vulnerability factor in one geographic area could be a resilience factor in another. In this case, being the parent or legal guardian of a child during the time of Sandy was associated with higher depression in Brooklyn, but lower depression in the Bronx.

Although further research in this area is needed to understand what factors account for geographic variation in vulnerability and resilience factors, there are several possibilities that could account for the findings. First, the strength of associations might depend on the distribution of both predictors and outcomes in the given area. For example, the risk associated with membership in a racial or ethnic minority group could depend in part on the proportion of residents in the group within a given neighborhood [20,21]. Along similar lines, other variables could influence the distribution, and risk associated with, a given factor. The presence of low quality housing or limited sheltering provision within a neighborhood [22], in combination with geographic exposure towards higher storm surges [19], for example, could shift the distribution of hurricane-related stressors and trauma (e.g., housing damage, displacement, bereavement) thereby strengthening their association with mental health outcomes. Second, the level of resources within a community could enhance or attenuate the influence of a vulnerability or resilience factor. For example, a lack of basic infrastructure or affordable and accessible health and childcare in the aftermaths of the disaster [23,24] might pose a higher risk for mental health problems for parents. Finally, geographic variation in more subtle cultural factors, such as informal social support networks and attitudes toward mental health service use, could influence associations between vulnerability and resilience factors and outcomes.

Few studies to our knowledge have explored geospatial variation in psychological vulnerability and resilience factors in the aftermath of disasters. Additional research is therefore needed to understand the mechanisms underlying our findings. This study, however, provides preliminary evidence that geography shapes both risk for adverse mental health outcomes and the predictors of such outcomes. This spatial epidemiological approach could be used to build resilience against disasters and other traumatic events, and to intervene in their aftermath. Pre-event interventions could build the psychosocial resources of demographic groups at greatest risk of adverse outcomes in a given neighborhood, for example. In the aftermath of events, a spatial epidemiological approach can be used to identify survivors at greatest risk and to plan for targeted interventions to reach them.

This study had some important limitations. First, although the sampling frame ensured that participants lived in neighborhoods that were exposed to Hurricane Sandy, this procedure likely limited the variance in hurricane-related stressors and traumatic events. It is possible that the pattern of results would have differed had we collected a representative sample for the entire NYC area. Nonetheless, the observation of substantial variability even in the context of a highly exposed population within affected areas (inundation zones) further underscores the variability that characterizes the spatial patterning of the mental health consequences of disasters.

Second, even though probability sampling was utilized, the results are unlikely to be fully representative of the population. For example, the results are unlikely to generalize to persons who have neither landline nor cellular telephones, or who are unlikely to answer calls from unfamiliar phone numbers. Hence, these likely differences between participants and non-participants may have limited the external validity of the study. Furthermore, although we detected few significant differences between complete and incomplete cases on the variables included in the study, there may have been additional unmeasured differences between them. Additionally, the non-response rate in the study was high and because the analysis was conducted at the person-level, we did not use sampling weights to account for non-response. However, prior research has shown significant differences in demographic characteristics and health status among survey respondents and non-respondents [25-27], and this issue further limits the generalizability of the study, with the potential that we might have underestimated levels of PTS and depression in the study area. Third, baseline prevalence of mental health (PTS and depression) was unavailable and as such, we do not know the extent to which mental health problems were longstanding, or whether they emerged after Hurricane Sandy. Even though depression and PTS symptoms were both assessed in reference to the prior month of the interview in our study and PTS was assessed with respect to the hurricane, prior research shows that pre-disaster mental health is among the strongest predictors of post-disaster mental health [11]. Hence, the symptom levels reported here could be conceptualized as reflecting both ongoing and emergent symptomatology. Fourth, rather low sample sizes within the inundation zones in each borough prevented us of looking at variation in vulnerability and resilience factors at a more detailed level of disaggregation. Given that the spatial clusters of above and below average mental health symptoms were found within borough neighborhoods, investigating spatial variations of psychological vulnerability and resilience factors at a finer spatial regimes level, such as the NYC community districts, could provide greater insight into the factors driving the spatial clusters of low and high PTS and depression. Fifth, our set of vulnerability and resilience factors was not comprehensive. For example, we used two items to screen for probable trauma exposure in addition to Hurricane Sandy in order to keep the questionnaire brief. Subsequent studies should use a more comprehensive list of traumatic events such as from the Diagnostic Interview Schedule (DIS) [28]. Furthermore, we did not assess perceived social support in the neighborhood, which is a robust predictor of post-disaster mental health [29] and, as such, should be included in future studies employing spatial epidemiological methodologies. Finally, the results are specific to the NYC and Hurricane Sandy context and, as such, we would not expect them to generalize to other geographic regions or to the aftermath of other disasters and mass traumatic events.

## Conclusions

This study demonstrated how a spatial epidemiological approach could be used to identify (1) locations with clusters of mental health problems after natural disasters, and (2) psychological vulnerability and resilience factors that are specific to a given region. This approach could be applied also to other regions around the globe. Especially with regard to a changing climate, more spatial epidemiological evidence is needed to build resilience against future mass traumatic events, and to identify mental health needs and targets for intervention in their aftermath in NYC and other areas worldwide.

## Methods

### Study design, sample, and data collection

We assembled a structured questionnaire in English and Spanish and collected self-report data from Sandy-affected New York City residents through telephone interviews between December 2013 and March 2014 (13–16 months post-disaster). The survey was designed to be 20–25 minutes to reduce the burden on participants and to increase the response rate and therefore required to be concise in regard to the items included. First, standardized inventories of mental health symptoms (PTS and depression) were included, since a primary goal of this study was to analyze mental health in Sandy-affected communities. Second, individual-level factors were gathered to reflect participant demographics and hurricane-related exposures found relevant in existent literature [9,10,30]. Furthermore, prior trauma exposure was assessed since previous research has found this to also be a robust predictor of post-disaster mental health [11].

**Figure 3:**
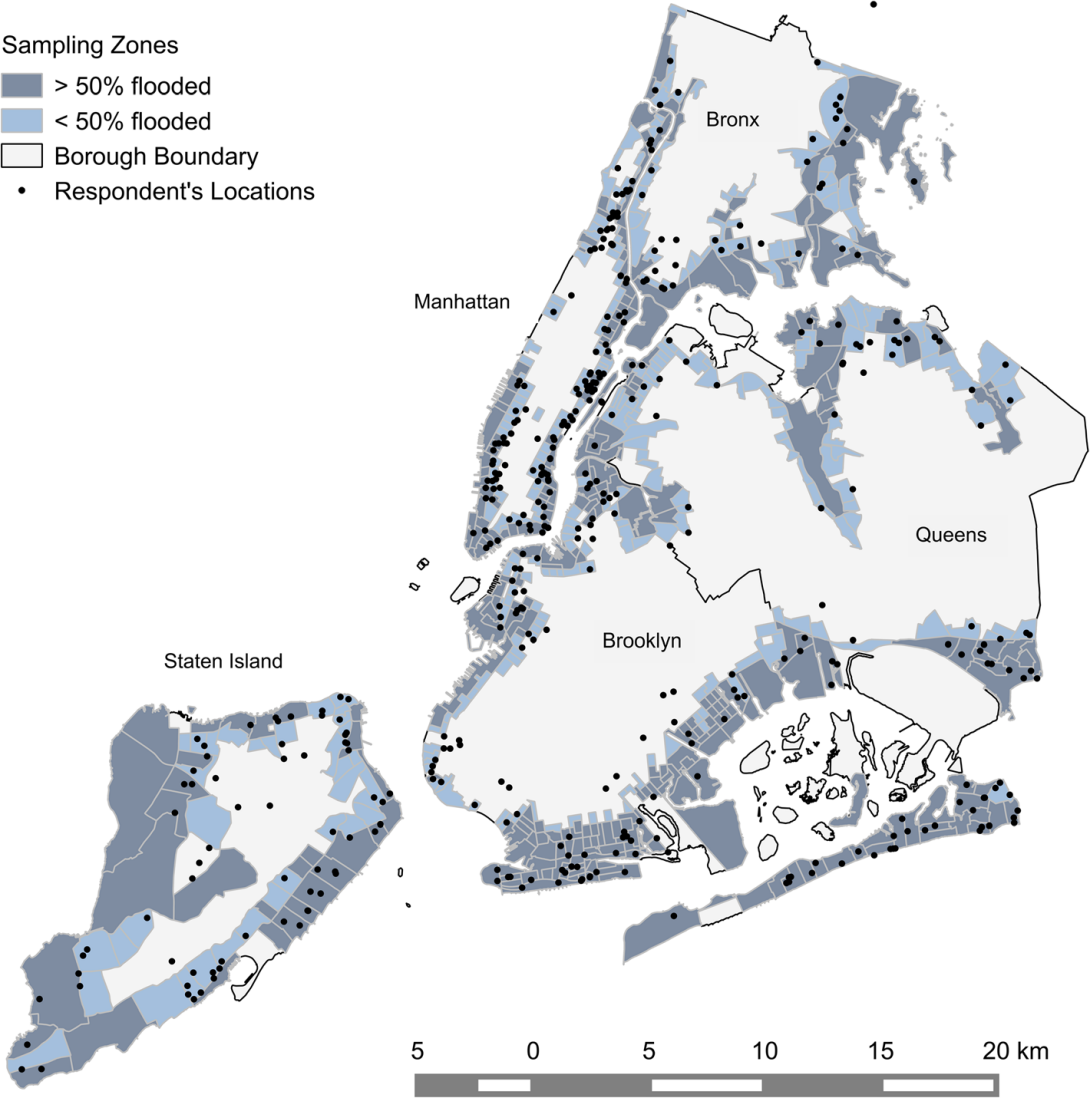
Distribution of respondents within sampling zones across NYC boroughs. Note that a geometric shift of approx. 100 m has been introduced for displaying the point data so that single houses may not be identified for privacy reasons.

Only adults (18 years and above) who lived in Sandy-affected flood inundation zones were considered eligible for our sample. The sampling frame was designed based on flood inundation data from the FEMA Modeling Task Force (FEMA MOTF). Zone 1 consisted of census tracts in which either 50% or more of the area was inundated and Zone 2 consisted of census tracts in which some, but less than 50% of the area was inundated and/or that were adjacent to the tracts from Zone 1 (cf. Figure 3) [4].

We combined a conventional sampling procedure with a random-digit dialing sampling in a novel approach to also capture the increasing number of residents that do not have a landline telephone. Within each zone, address-based sampling was applied to half of the sample, for which household members were enrolled via mail or landline telephones and one adult from each household was randomly selected for the interview. The other half of the sample within each zone was enrolled via random-digit dialing of mobile phones. Within this group, we only considered the person answering the phone as eligible for participating in the interview since mobile phones are, unlike landline telephones, attached to a person rather than to a household. Mobile phone participants were asked for their zip codes as a geographic screening to determine whether they likely resided in the sampling zones. Participants provided their mailing addresses upon interview completion, and it was then determined that a small percentage lived outside of the sampling zones at the time of the hurricane (10.0%; n = 42), but were included in the analysis nonetheless. We attempted to reach potential respondents up to 15 times, and achieved an overall response rate of 35%, which is consistent with similar population-based post-disaster studies [31,32]. A total of 500 participants completed the survey; 453 provided pre-hurricane addresses, and 35 of these were excluded due to missing data. A series of Bonferroni-correct independent samples t-tests and chi-square tests found that participants included in the analysis were significantly more likely to be female and were significantly older, compared to those dropped due to missing data. The final sample consisted of 418 participants merely living in affected areas within five boroughs. After the study was described to participants, oral consent from participants was obtained. Oral informed consent was employed instead of written informed consent because all interviews were conducted over the telephone. Interviewers documented participants’ informed consent in the study database. This approach to obtaining informed consent, as well as all other study procedures, was approved by the institutional review board of Columbia University.

### Disaster-related posttraumatic stress (PTS)

We defined Hurricane Sandy-related posttraumatic stress (PTS) symptoms according to the Diagnostic and Statistical Manual of Mental Disorders, Fifth Edition (DSM-V), and measured PTS with the Posttraumatic Stress Checklist for DSM-V (PCL-5) [33]. On this checklist, participants rated the extent to which they were bothered by each symptom (e.g., “repeated, disturbing, and unwanted memories of Hurricane Sandy”) in the past 30 days from 0 (not at all) to 4 (extremely). The responses were summed to obtain a severity score, ranging from 0 to 80. Although the psychometric properties of the PCL-5 are under examination, the DSM-IV version of the scale was shown to have excellent internal consistency and substantial agreement with PTSD diagnosis and symptom ratings [34]. Cronbach’s alpha of the PCL-5 in our study was 0.93. For similar measures of posttraumatic stress albeit with a different checklist, telephone interviews were previously found a reliable method of interviewing [35].

### Depression

We assessed depression with the nine-item Patient Health Questionnaire (PHQ-9) [36]. Depression items were general – that is, without the phrasing “due to the hurricane and its aftermath” – and may therefore reflect both ongoing and post-disaster depression. Participants indicated how often over the past 30 days they had been bothered by each symptom (e.g., “feeling down, depressed, or hopeless”) from 0 (not at all) to 3 (nearly every day), which resulted in a severity score as the sum of all items, ranging from 0 to 27. The PHQ-9 has excellent internal consistency, test-retest reliability, and construct validity as found in other studies [37]. Cronbach’s alpha of the PHQ-9 in our study was 0.88. For depression, phone interviews have been found to be a reliable interviewing method [38].

### Explanatory variables

Participants provided individual-level demographic information, such as their age, gender, and race/ethnicity (cf. Table 1). For socioeconomic characteristics, we asked for their highest level of education and employment status at the time of the interview. Participants also reported on their living arrangements, including their marital status at the time of the interview, and whether they were the parent or legal guardian of a child under 18-years old and living with this child at the time of the hurricane. To assess probable trauma exposure in addition to Hurricane Sandy, we included two screening questions. Specifically, we asked (1) “Not including things that happened during the storm, did something terrible ever happen to you so that you thought you might get hurt very badly or killed?”, and (2) “And again, not including things that happened during the storm, did you ever, in your life, see anything terrible happen to someone else so that you thought they might get hurt very badly or killed? Participants who answered “Yes” to either question were classified as having experienced a traumatic event in addition to the hurricane.

To assess Sandy-related exposure, we used scales that were successfully applied in other epidemiological surveys on mental health in the aftermath of major hurricanes [10,39]. Hurricane Sandy-related stressors included whether participants (1) were displaced from their pre-hurricane home for over a week, (2) went without electricity, heat, or water for over a week, (3) experienced decline in income due to the hurricane and its aftermath, and (4) had damage to their pre-hurricane home. The four questions were summed up for an affirmative response count for Hurricane Sandy-related stressors. Hurricane Sandy-related traumatic events included whether the respondent (1) was personally injured, (2) had a close friend or family member who was injured, and (3) had a close friend or family member who was killed, each as a direct result of the hurricane and its aftermath. Again, a sum of affirmative responses was included. Table 1 includes descriptive statistics for all study variables, both for the full sample and for subsamples residing in each borough at the time of the hurricane.

### Analytical methods

#### Global and local spatial autocorrelation analysis

We were interested in the geographic variability of Sandy related mental health and related psychological vulnerability and resilience factors at the person-level unit of analysis. Therefore, we utilized an unweighted dataset, and conducted spatial autocorrelation analysis to test for spatial clusters of PTS and depression based on geocoded addresses of respondents.

Global spatial autocorrelation is characterized by the degree to which geographically close respondents tend to have similar or dissimilar levels of a given outcome [40]. We assessed univariate global spatial autocorrelation for PTS and depression by using the global Moran’s I statistic. This statistic gives a formal indication of the linear association between observed values and the weighted average of neighboring values, i.e. the spatial lag [41]. Generally, the Moran’s I ranges from −1 (dispersed pattern) over 0 (random) to +1 (clustered), but is not necessarily bound to these values depending on the neighborhood weights matrix used. We tested for two separate neighborhood definitions (the k-nearest neighbors (k-nn) and fixed distance bands). We used a row standardized neighborhood weights matrix to create proportional weights for features that had an unequal number of neighbors.

The 4, 8, 12, and 16 nearest neighbors were tested first, which had maximum distances of 5.7 km, 7.9 km, 8.4 km, and 10.1 km between the neighbors, respectively. We further applied an incremental spatial autocorrelation test based on fixed distance bands. To ensure that every respondent had a neighbor, we started with the value of the largest distance between two nearest neighbors (3.5 km) and incrementally increased this value by 0.5 km up to distances of 17.5 km.

Local autocorrelation analysis was used to identify significant spatial clusters of observations (similar neighbors) or spatial outliers (dissimilar neighbors). The local Moran’s I was calculated as a local indicator of spatial association (LISA) [42]. Respondents’ locations with significant (p < 0.05) local Moran’s Is were classified into High-High or Low-Low clusters (mental health outcome above or below average surrounded by a spatial lag above or below average, respectively), and High-Low or Low-High spatial outliers (mental health outcome above or below average surrounded by a spatial lag below or above average, respectively). Those neighborhood definitions that were found significant and obtained the highest global Moran’s I and z-values for both PTS and depression were used for the local Moran’s I statistic. Significance of the global and local Moran’s I were calculated using a randomization test on the z-value with 9,999 permutations [43]. Incremental global spatial autocorrelation analysis was done in ArcGIS, version 10.2 [44], all other spatial autocorrelation analysis was applied in GeoDa [45,46].

### Spatial regimes regression

To test for variations in psychological vulnerability and resilience factors in flood zones across the five boroughs, we applied a spatial lag regression approach with regimes, since a preceding analysis with geographically weighted regression (GWR) showed marginal spatial variation of model coefficients for both PTS and depression (results available upon request). Boroughs were chosen as the geographic unit for these analyses, since we assumed that there would be greater variability in vulnerability and resilience factors, as well as in PTS and depression, in these larger regions than within census tracts or community districts (cf. Table 1).

We first fitted a multivariable linear regression model based on ordinary least squares (OLS) to account for global associations between the explanatory variables and mental health outcomes, ignoring a likely variability across the boroughs. Second, we fitted an OLS model with the boroughs included as spatial regimes. This allowed us to model group-wise heteroskedastic errors, meaning a different error variance as well as different betas for each of the boroughs. Significantly different slope coefficients between boroughs would suggest that the relationship of the mental health outcomes with the explanatory variables was heterogeneous, meaning that there was significant variation across the boroughs. Third, spatial autocorrelation analysis was applied on the OLS residuals from the model with regimes to guarantee that assumptions of independence among model coefficients would not be violated. For example, the independence assumption would be violated if we omitted an explanatory variable that also varied spatially, resulting in spatially dependent model residuals [40]. We assessed residual spatial autocorrelation with the Anselin-Kelejian [47] and Lagrange multiplier tests [48]. Fourth, a spatial lag regression model with regimes was fitted by including a fixed spatial lag coefficient to control for a likely spatial structure in model residuals. The spatial lag was a weighted average of neighboring values of the given mental health outcome and was used to account for mismatches in scale effects between the person-level unit of analysis and the area-scale of the impact, that could arise for example from those areas closer to the water with assumable higher exposure to the hurricane.

Model estimates were based on spatial two stage least squares estimation [49,50]. For the regimes models, we used the same neighborhood matrix as for the univariate global and local spatial autocorrelation analysis. Fifth, to assess the degree of variation among the boroughs, we applied the Chow test on structural instability [51]. The null hypothesis of this test is spatial homogeneity, or equality of the coefficients across the boroughs; the alternative hypothesis is spatial heterogeneity, or that some or all of the coefficients vary across the boroughs. We applied all spatial regimes regressions and respective tests in GeoDaSpace [52].
